# Imaging individual protein aggregates to follow aggregation and determine the role of aggregates in neurodegenerative disease

**DOI:** 10.1016/j.bbapap.2018.12.010

**Published:** 2019-10

**Authors:** Suman De, David Klenerman

**Affiliations:** a Department of Chemistry, University of Cambridge, Lensfield Road, Cambridge CB2 1EW, UK; b UK Dementia Research Institute, University of Cambridge, Cambridge CB2 0XY, United Kingdom

## Abstract

Protein aggregates play a key role in the initiation and spreading of neurodegenerative disease but have been difficult to study due to their low abundance and heterogeneity, in both size and structure. Fluorescence based methods capable of detecting and characterising single aggregates have recently been developed and can be used to measure many important aggregate properties, and can be combined with sensitive assays to measure aggregate toxicity. Here we review these methods and discuss recent examples of their application to determine the molecular mechanism of aggregation and the detection of aggregates in cells and cerebrospinal fluid. The further development of these methods and their application to the aggregates present in humans has the potential to solve a major problem in the field and allow the identification of the key toxic species that should be targeted in therapies.

## Highlights

• Individual protein aggregates can be imaged using fluorescence imaging.
• Ultra-sensitive assays have been developed to measure aggregate toxicity.
• The aggregation mechanism of proteins can be determined.
• Experiments can be performed in cells or human cerebrospinal fluid.
• These methods can potentially identify the toxic aggregates that cause neurodegenerative disease.

## 1 Introduction

The assembly of protein monomers, into small soluble aggregates (oligomers), to ultimately form insoluble fibrils is fundamentally important. It is a process that can occur for most proteins, under suitable conditions, and it also plays a major role in the initiation and spreading of protein aggregates through the brain in neurodegenerative diseases such as Alzheimer's and Parkinson's disease [1]. Studying this process in the test-tube, in cells or in humans, is challenging since only a small fraction of the protein forms oligomers at any given time; typically <1%. These oligomers are heterogeneous in both size and structure unless strategies are used to enrich one oligomeric species [2,3]. Furthermore, it is important to be able to relate the structure of the aggregates to their properties, particularly biologically relevant properties such as cytotoxicity or ease of degradation. Some aggregates may not be toxic at all, while others of a specific structure and size might be highly toxic. Approaches capable of imaging and measuring the properties of individual aggregates have been developed to address these problems and will be discussed in this review.

While it is possible to perform controlled aggregation reactions of proteins in the test-tube, such as the aggregation of alpha-synuclein (αS) associated with Parkinson's disease and amyloid-β (Aβ) and tau associated with Alzheimer's disease (AD), extrapolation of this data to what happens during the course of disease is challenging. This is because it can take decades to develop these diseases compared to the hours to few days to perform the experiments, creating a time gap. This means that it is important to study both the kinetics and thermodynamics of the aggregation process, since it is not clear in which regime the disease process occurs. Furthermore, the test-tube experiments are generally performed at concentrations that are higher than those *in vivo* by several orders of magnitude, so one needs to be able either to perform experiments at physiological concentrations, picomolar in the case of Aβ oligomers, or to extrapolate back these conditions. In addition, during the aggregation process *in vivo*, the monomers are also modified by, for example, truncation or phosphorylation so that the fibrils formed consist of largely or entirely chemically modified monomers. Thus there are three gaps that need to be bridged between test-tube and *in vivo* experiments: a time, concentration and reality gap.

One strategy to deal with these issues and bridge these gaps is to develop methods that can directly image and characterise the individual protein aggregates in human samples. Another strategy is to develop kinetic and thermodynamic models of the aggregation process, which fit the experimental data obtained at higher concentrations, and then extrapolate this data to longer times and lower concentrations that are not accessible experimentally. This can be combined with a bottom-up approach where studies are performed on systems of increasing complexity; such as in the studying the aggregation of post-translational modified proteins or performing experiments in the presence of two proteins or in the presence of chaperones, which are present *in vivo* and act to inhibit aggregation. Given the large number of possible variables, these experiments need to be informed by the variables that have been shown to be important in animal models and humans.

There have been recent reviews of single molecule studies of protein aggregation [4,5]. This review will focus on what can be learnt from studies of protein aggregates at the level of individual aggregates using fluorescence, and the advantages and disadvantages of this approach. It will critically discuss what we have learnt to date about protein aggregation and neurodegenerative disease, and what needs to be done in the future to gain new insights into the role of protein aggregates in neurodegenerative disease in humans.

## 2 The single aggregate approach

Single protein aggregates can be detected by fluorescence-based methods, atomic force microscopy or electron microscopy. This review will focus only on fluorescence based methods. The main concept behind single molecule fluorescence is that a single fluorophore either covalently or transiently bound to the molecule of interest can emit 1-10 million photons before photobleaching, so that a detectable fluorescence signal can be generated from one fluorophore on a single protein molecule. If transient binding is used, it is possible to observe molecules for long periods of time, which opens up the possibility to do biochemical reactions on individual molecules.

**Fig. 1:**
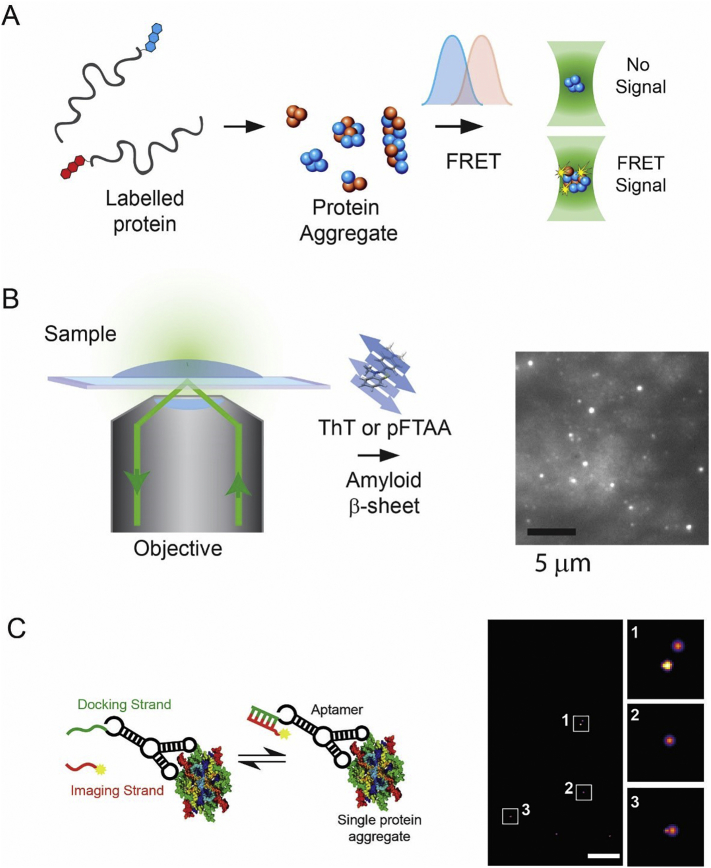
**Principles of single aggregate measurements**. A) Fluorophore labelled monomer can undergo intermolecular FRET between a donor and acceptor fluorophore when aggregates are formed. This allows aggregates to be distinguished from monomer. Detection of coincident red and blue fluorescence allows the number of aggregates to be counted and their relative size to be estimated by comparing the fluorescence intensity to that of the monomer. The extent of FRET can also be measured which provides information about the compaction of the aggregate and if one or multiple species are present. B)Total internal reflection fluorescence microscopy can be used to detect aggregates on a glass coverslipusing the transient binding nature of dyes such as Thioflavin T or the pentameric form of formyl thiophene acetic acid (pFTAA), whose quantum yield increases significantly when bound to beta sheet structures in the aggregates. C) The transient binding of a fluorophore labelled DNA imaging strand to the docking strand on an aptamer that binds aggregates allows single molecule localisation to be performed. This enables aggregates of Aβ to be imaged at super-resolution. The scale bar is 500 nm.

Fluorescence based methods can be performed on aggregates in solution or immobilised to a surface (Fig. 1). Solution based methods, generally using a confocal microscope set-up, avoid any bias in surface affinity but provide shorter observation times (<1 ms) while surface based methods, generally using a total internal reflection fluorescence (TIRF) microscopy set-up allow longer observation times (seconds to hours). There are several different strategies to distinguish monomer from oligomers. This can be done by labelling each monomer with the same fluorophore and distinguishing monomer from oligomer based on fluorescence intensity or the number of photobleaching steps [6], since oligomers should have multiple photobleaching steps. A method with higher discrimination is to label monomers with either red or blue fluorophores. Oligomers can be detected by the presence of both fluorophores in the same spatial region, when imaging, or the emission of simultaneous red and blue fluorescence if performed in solution [7,8]. This method can be extended to detect intramolecular Fluorescence Resonance Energy Transfer (FRET) between a donor and acceptors fluorophore on different monomers in the same oligomer [9] (Fig. 1A). The extent that FRET occurs can also provide information about the structure of the oligomer in certain cases.

An alternative strategy is to use molecules that specifically bind to oligomers of certain structure or composition. These can be antibodies, nanobodies or aptamers either labelled with a dye or which have a DNA strand attached (Fig. 1B and C). Transient binding of the complementary imaging strand to the DNA on the antibody allows imaging of the aggregate over long periods of time, a method called DNA PAINT (point accumulation for imaging in nanoscale topography) [10]. Alternatively, dyes like Thioflavin T can be used, that have significantly higher affinity for aggregates than monomer [11]. Thioflavin T reversibly binds to oligomers with beta sheet structure. Moreover, several of the molecules developed for Positron-emission tomography (PET) imaging of protein aggregates in the human brain are also fluorescent and can also be used for aggregates imaging [12].

### 2.1 What can be measured?

In the case when the monomer is labelled with a fluorophore, the first thing that can be measured is the fraction of all monomers that form oligomers; hence the concentration of oligomers can be determined. It is also possible to get information about relative oligomer size, compared to a monomer, from the intensity of the fluorescence signal. This is the apparent oligomer size since it is estimated from the fluorescence signal and relies on there be no significant fluorophore quenching in the oligomer. The extent of FRET (FRET efficiency) can also provide information about oligomer structure, particularly if aggregates with different structures are present [9]. In the case when specific oligomers are detected using molecules that transiently bind, then the number of these oligomers can be determined, although here calibration samples will be needed to convert this into a fraction or concentration.

It is also possible to work in a regime where only a single fluorophore is bound to an oligomer at any time. In this case it is possible to localise the position of this fluorophore to 20–40 nm depending on the intensity of the signal. Multiple rounds of imaging of individual aggregates then allows images of the aggregate to be obtained at 20–40 nm resolution allowing their size to be determined. This approach can be used for both surface immobilised aggregates and can also be performed in fixed cells [13].

### 2.2 Obtaining more information about aggregates

As well as measuring the number and concentration of the aggregates in a sample, it is possible to obtain a great deal more information. In general, samples need to be diluted to picomolar concentrations for single molecule experiments. Unstable species can dissociate in this dilution step so if performed rapidly, using microfluidic devices, it is possible to measure these unstable species [14] and also detect species more rapidly [15]. However, it is probably the species that are stable at these low concentrations that are more relevant to the disease pathogenesis, given the concentration of oligomers is low *in vivo*, for example the concentration of Aβ42 oligomers is 1–10 pM in human (CSF) [16]. Dilution into buffers of high salt or low salt to measure the stability of the oligomers and monitoring their stability over time is a strategy that can be used to measure their relative stability and hence resolve different species. It is also possible to add enzymes such as proteinase K (PK) and then measure which oligomer remain and which are degraded after a certain time, to distinguish oligomers based on their PK resistance.

More information is also available from dyes that bind transiently to the aggregates. Use of environmentally sensitive dyes allows one to measure the surface hydrophobicity of aggregates [17,18]. For example, Nile red's fluorescence spectrum shifts depending on its hydrophobic environment. Since this dye also binds transiently to aggregates it can be used to simultaneously measure aggregate size and surface hydrophobicity. The extent of modulation of the dye fluorescence can also be used to probe the extent that the aggregate structure is ordered and distinguish fibrillary aggregates [19].

## 3 Oligomer properties

Obtaining information about the number, size and structure of the oligomers present in samples is important. However, some oligomers might be highly toxic to cells and some might be non-toxic, so it is vital to have sensitive assays to measure parameters associated with oligomer toxicity and the interaction of oligomers with putative receptors. Currently there are no assays sensitive enough to measure the toxicity of a single oligomer, so experiments need to be performed on a solution where the oligomers have been characterized and the toxicity is measured and then a second sample, where the number or structure of the oligomers is altered, is used in order to determine which species are more toxic.

Since the physiological concentration of oligomers is in the low picomolar range it is important to develop assay that work in this concentration range. An important point is that the gap between cells is only about 50 nm in the brain [20] so that the volume round a cell 10 μm in diameter is only 15 fL and contains 0.01 molecules at an oligomer concentration of 1 pM. The oligomer concentration needs to be 100-fold higher than physiological levels for there to be one oligomer per cell. Even at 20 nM oligomers, the estimated concentration of Aβ oligomers above a fibril [21], there are only 200 oligomer per cell and not all of these oligomers will bind to receptors in the cell membrane. Therefore, even if the local concentration is higher than physiological levels, these estimates suggest that toxic effects will need to be mediated by single oligomers, certainly at the start of the disease. This means that the effect of the oligomers needs to be caused by binding a single receptor or occur via mechanism that does not involve specific binding to receptors. In contrast, for experiments performed *in vitro*, at a fixed oligomer concentration there is a much larger volume of oligomers per cell and hence many more oligomers can interact with a cell over extended times causing effects that might not occur under more physiological conditions.

**Fig. 2:**
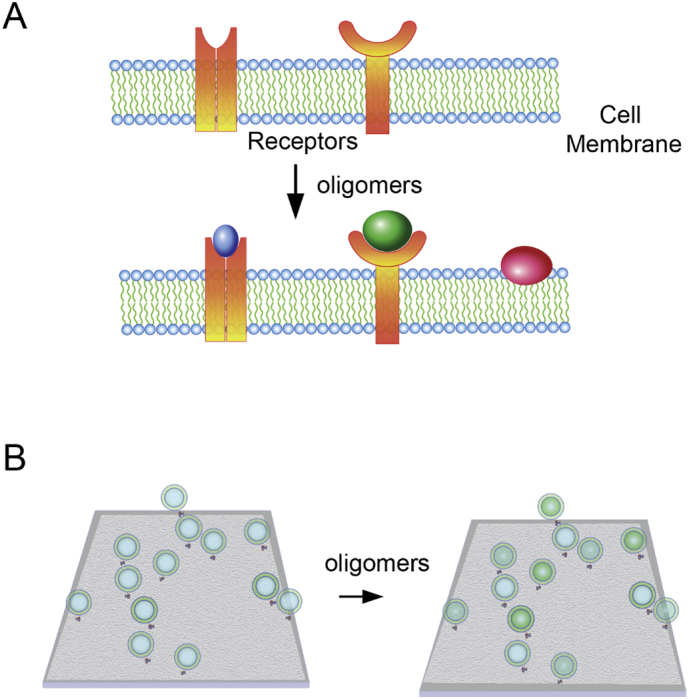
**Oligomer toxicity** A) Different size aggregates can bind receptors in the cell membrane leading to signalling. This can be detected by imaging if the receptor and aggregate are both fluorophore labelled since the fluorescence signals will co-localise. Alternatively, aggregates can bind non-specifically to the cell membrane which leads to a fluorescence signal that does not co-localise with the labelled receptor and can induce membrane permabilisation. B) A liposome assay using small surface immobilised liposomes, containing a Calcium binding dye can sensitively detect membrane permeabilisation by oligomers. Calcium ion entry from the bath into the liposome leads to increased fluorescence in the liposome that can be sensitively detected by total internal reflection fluorescenece microscopy.

Using sensitive imaging techniques, it is possible to directly observe the binding of fluorophore labelled oligomers to fluorophore labelled receptors and hence directly determine the extent of binding as a function of oligomer concentration. This is important since in some cases oligomers may effect receptor function by indirect mechanisms rather than directly binding to the receptor. For these experiments to be possible the density of the receptor on the cell surface needs to be 1 receptor per μm^2^ or lower so that individual receptors can be visualised using diffraction limited imaging (Fig. 2A). In principle higher receptor densities could be studied using super-resolution imaging, although this has not been done to date to our knowledge. The receptor needs to be labelled in a manner that does not significantly perturb function using antibodies or genetic encoding of the label. Despite the potential of this method to directly measure the affinity of oligomers for putative receptors in the cell membrane, so far it has only been applied to the interaction between Aβ oligomers and prion protein [22].

We recently developed an ultra-sensitive assay to measure the ability of oligomers to permeabilise a lipid membrane to allow the entry of calcium ions [23] (Fig. 2B). Calcium dysregulation is commonly observed in neurodegenerative disease, so this assay provides one measure of oligomer toxicity. The assay is based on thousands of liposomes arrayed on a surface. Aggregates added in solution can permeabilise the lipid membrane, resulting in calcium ions from the bath entering the liposome. Calcium ion entry is detected by the presence of a calcium ion binding dye present in the liposome using fluorescence imaging in a TIRF microscopy mode. Generally, there is only a small increase in calcium ions showing that the aggregates do not form permanent pores in the membrane. At the end of the experiment ionomycin is added to measure the intensity of the fluorescence signal when each liposome is filled calcium ions. This assay allows the sensitive quantification of the number of lipid membrane permeabilising aggregates present in the sample. This is based on averaging the measurements from many individual liposomes and we have shown that it can detect oligomers at concentrations as low as 1 pM [23]. This assay can potentially be made more sensitive and selective by optimising the lipid composition for the oligomers of interest.

A related assay has recently been developed that uses giant plasma membrane vesicles which detects both the binding of oligomers of amylin to the vesicle membrane and entry of green fluorescent protein into the vesicle [24]. FRET with low numbers of donor and acceptor labelled monomer was used to show that the oligomers that cause permeation are dynamic, contain hundreds of monomers and cause permeation by two mechanisms. Using enriched αS oligomers a recent study using NMR showed that the enriched oligomer species that was most effective at membrane disruption was lipophilic, to facilitate membrane binding, and had rigid beta-sheet structure that inserted into the membrane to disrupt its structure [25]. This study provides new insights into the molecular basis of oligomer induced toxicity.

## 4 Limitations of current methods

Since the aggregates exist in a range of different sizes and structures one issue is whether all oligomers are detected in any given study. Only if experiments are performed with dye labelled protein can one be confident that all aggregates present are detected and this is still only the stable oligomers that are still present at dilution to picomolar concentrations for single molecule imaging. However, these stable aggregates are arguably the most relevant under a physiological setting. For unlabelled aggregates one approach would be to use a range of aggregate specific reagents to ensure the majority of aggregates are detected. Sizing using fluorescence intensity is only approximate and the lack of model samples of known stoichiometry makes it difficult to calibrate the method, so direct imaging of the aggregates using super-resolution to determine their size is a better approach. AFM has recently been demonstrated to also be a useful method to more accurately measure aggregate size and follow dynamics [[26], [27], [28]], so use of high resolution AFM would nicely complement fluorescence based methods. Expanding the number of measurements that can sensitively determine properties of the aggregates related to their cellular toxicity would also be highly desirable, especially on cells. Sensitive assays have been developed to detect the production of cytokines during T-cell triggering. Since the same cytokines are also produced during neuroinflammation, it would be interesting to explore application of these methods to aggregate induced inflammation [29].

## 5 What are the key questions?

Before reviewing what has been done using these methods it is useful to discuss the following issues: what are the key questions that need to be addressed; what questions can currently be addressed and what advances are needed to bridge the gap between these two sets of questions? In our view, the key questions are which are the aggregates responsible for the initiation and spreading of neurodegenerative diseases through the human brain? By what mechanism do aggregates form from monomer in the brain? By what molecular mechanisms do they damage and kill neurons? By what mechanisms do they spread from neuron to neuron? Which are the key species that should be targeted in therapy?

So far the questions that have been addressed to a greater or lesser extent by single aggregate studies are what is the molecular mechanism for aggregation of pure proteins in the test-tube; which species are formed and which are toxic and by what mechanism? Such experiments provide a baseline for the range of protein aggregates that might be formed *in vivo* and their potential mechanisms of toxicity. However, they fail to deal with the potential complexity *in vivo* where there are significant post-translational modifications and there are different unfolded proteins present that can potentially interact with each other over time [30,31]. This in turn highlights the need to develop methods to detect and characterise the real protein aggregates present in humans with neurodegenerative diseases such as Alzheimer's and Parkinson's disease.

## 6 Examples of studies of single aggregates to date using fluorescence methods

Since single aggregate studies have been comprehensively reviewed very recently [4], we will go through some recent examples of the different types of experiments that have been performed done from our own work, and discuss what insights they provide into the questions above.

### 6.1 The aggregation pathway for αS

The protein αS self-assembles into small oligomeric species and subsequently into amyloid fibrils that accumulate and proliferate during the development of Parkinson's disease. We labelled αS at position 90 close to the NAC region of the protein with either a donor or acceptor fluorophore [9]. Oligomers could be detected selectively detected by FRET between the donor and acceptor fluorophores in an oligomer and we found that distinct species with high and low FRET could be detected. The low FRET species was sensitive to PK degradation while the high species was PK resistant.

**Fig. 3:**
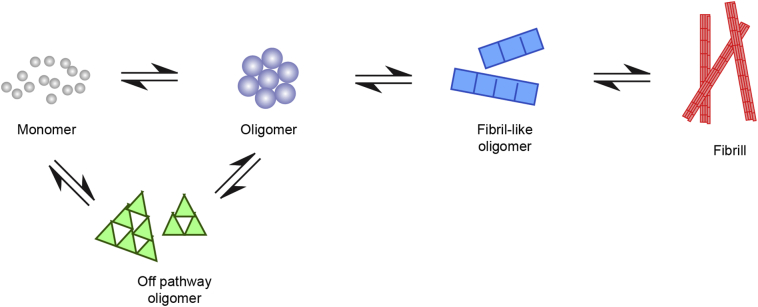
**General pathway for aggregation of proteins to form amyloid fibrils.** Monomers form oligomers which undergo a slow structural conversion to fibril-like oligomers which then can grow by monomer addition into long fibrils. Monomers can also assemble into off-pathway oligomers. The relative number of the different species depends on the forward and backward rate constants and can be modified by the addition of chaperones or antibodies.

We performed experiments over a range of monomer concentrations from 0.5 to 140 μM and found that the data could be fitted to a kinetic model with slow unimolecular conversion steps between low FRET (globular) oligomers and high FRET compact oligomers [32]. These compact oligomers converted to fibril-like oligomers which then went onto form fibrils by monomer addition (Fig. 3). We found that the high FRET species were significantly more effective at inducing production of reactive oxygen species in neuronal cells than the low FRET species. We could use this kinetic model to predict the effectiveness of aggregate seeds at inducing aggregation when entering a cell, assuming that the cell just acted as a container of αS molecules. In this case a large number of seeds needed to enter a cell in order for the rate of aggregation to be enhanced. Since smaller number of aggregates could induce production of ROS this suggested that cell stress may contribute to spreading. However, if nucleation was prevented in the cellular environment, so that no aggregation occurred then a single seed would be able to induce aggregation of the αS in a cell. Experiments with Nile red imaging of the surface hydrophobicity also showed that the oligomers are structurally distinct from fibrils, being more hydrophobic which in combination with their smaller size probably contribute to their toxicity [17].

Overall, these experiments showed that there is rapid formation of globular oligomers that then slowly convert into more toxic and PK resistant beta-sheet rich oligomers. These in turn grow and convert into fibril-like oligomers, which can then go onto form fibrils. In contrast to the millisecond to second times associated with protein folding, the time scale for these key conversion steps is 10 h.

We extended these studies to study the aggregation of pathological mutants of αS [33]. Certain missense mutations in the gene encoding for αS induce early-onset forms of the disease. It has been suggested that these variants might have an inherent tendency to produce high concentrations of oligomers during aggregation, although a direct experimental evidence is still missing. We compared wild-type αS to A53T, A30P and E46K mutants and compared both the aggregation kinetics and the structural properties of the ensemble of oligomers generated. We found that the kinetics of oligomer formation correlates with the natural tendency of each variant to acquire beta-sheet structure. Moreover, A53T and A30P showed significant differences in the averaged FRET efficiency and only one of the two types of oligomers formed compared to the wild-type oligomers. Importantly, we found similar concentrations of oligomers during the lag-phase of the aggregation of wild-type and mutated αS, suggesting that the properties of the ensemble of oligomers generated during self-assembly might be more relevant than their absolute concentration for triggering neurodegeneration. Other properties of the oligomers that might be more important such as their structure, stability or removal rates.

### 6.2 Tau aggregation

We also used single-molecule fluorescence to study the aggregation of the repeat domain of tau (K18), which forms the core region of neurofibrillary tangles found in AD. Our initial kinetic analysis reveals that aggregation proceeds via monomeric assembly into small oligomers, and a subsequent slow structural conversion step before fibril formation [34]. Using this approach, we have been able to quantitatively determine how specific disease associated mutations alter the aggregation energy landscape. More recently, we extended these studies and distinguished several subpopulations of oligomers with different stability, by diluting into buffers with different ionic strength [35]. We followed the evolution of these different oligomeric species during aggregation reactions as a function of temperature and concentration.

Employment of techniques from chemical kinetics reveals that the two largest populations are structurally distinct from fibrils and are both kinetically and thermodynamically unstable. The first population is in rapid exchange with monomers and held together by electrostatic interactions. The second is kinetically more stable, dominates at later times, and is probably off-pathway to fibril formation. These more stable oligomers may contribute to other oligomer induced effects in the cellular environment, for example, by overloading protein quality control systems. We also showed that the shortest growing filaments remain suspended in aqueous buffer and thus comprise a third, smaller population of transient oligomers with cross-β structure. Our data showed that a diverse population of oligomers of different structures and half-lives are formed during the aggregation reaction with the great majority of oligomers formed not going on to form fibrils.

### 6.3 Generality of protein aggregation mechanism

Our studies of αS and tau show that distinct oligomeric species are formed, and a conversion step is needed to form fibril-like aggregates. A recent study of the yeast prion protein Ure2 demonstrates the formation of two separate oligomer species with different kinetic and structural properties [36]. Both oligomers are unstable and predominantly dissociate and are on-pathway to fibril formation. This is very similar to the aggregation mechanism for αS but with the formation of oligomeric species with decreased stability.

One issue with the study of tau aggregation is the need to induce aggregation using heparin, unlike in cells where it can occur spontaneously resulting in highly phosphorylated tau fibrils. While it is possible that some of the species are observed due to the use of heparin, these experiments also highlighted that the most frequently occurring oligomers are transient in nature. A significant energetic rearrangement would be needed for these to convert to growth-competent, fibril-like oligomers, which are present at far lower concentrations. This means that a wide range of oligomeric species can be present at the start of the aggregation process, with different properties, and only as the aggregation proceeds fibril and fibril-like oligomeric species become the dominant species. A wider range of species might be expected to be formed if the aggregation reaction occurs at lower concentrations and longer times than in these relatively short few hour experiments. There will be more time to explore more of the energy landscape although this regime has not been explored to date experimentally.

Both synuclein, tau and Ure2 show a generic mechanism with the formation of oligomers that convert into fibril-like oligomers, with the possibility of the formation of off-pathway oligomers (Fig. 3). The relative rates of the forward and backward reaction determine whether the oligomers are transient or stable. While this is the mechanism in solution, it is likely to be modified by many of the molecules present in the cell. This could be either by changing the reaction rate constants or by adding additional pathways or the presence of additional surfaces such as lipid membranes.

### 6.4 Increasing complexity

Protein aggregation occurs in the complex environment of the cell, which contains lipid membranes and chaperones which can alter the rate and pathway of aggregation. To study the effect of a particular cellular component, one can perform experiments in the presence of different concentrations. We have done such experiments for chaperones and lipids known to be important in neurodegenerative disease.

It is proposed that αS forms soluble alpha-helical multimers in healthy neurons [37]. We made αS multimers in the test-tube using arachidonic acid (ARA), one of the most abundant fatty acids in the brain, and characterized them by a combination of bulk experiments and single-molecule FRET measurements [38]. The data suggested that ARA-induced oligomers are alpha-helical, resistant to fibril formation, more prone to disaggregation, enzymatic digestion and degradation by the 26S proteasome, and lead to lower neuronal damage and reduced activation of microglia compared to the oligomers formed in the absence of ARA. These multimers can be formed at physiologically-relevant concentrations, and pathological mutants of αS form less multimers than wild-type αS. Our work provides strong biophysical evidence for the formation of alpha-helical multimers of αS in the presence of a biologically relevant fatty acid, which may have a protective role with respect to the generation of beta-sheet toxic structures during αS fibrillation.

We also studied the effect of the extracellular chaperones Clusterin and α_2_-macroglobulin on the aggregation of αS, using one fluorophore on the chaperone and a different fluorophore on the αS [39]. We found that both chaperones tended to bind small oligomers (<5 αS monomers) in an approximate equimolar ratio. Oligomers containing >5 αS monomers were observed to be associated with proportionally less chaperone, suggesting that the number of chaperone-accessible binding sites on the αS oligomer surface does not increase linearly with the number of αS monomers in an oligomer. Clusterin exhibits differential binding to αS oligomers that may be related to structural differences between two previously described forms of αS oligomers, in particular the high hydrophobicity of smaller oligomers. The binding of both chaperones appears to shield the surface hydrophobicity present on the oligomers and reduces the ability of the oligomers to permeabilize lipid membranes and prevents an oligomer-induced increase in ROS production in cultured neuronal cells.

A similar study investigated the binding of the chaperone heat shock protein 70 (Hsp70) to tau aggregates [40]. We found that Hsp70 has a much higher affinity for oligomers and fibrils, than monomer, with the affinity increasing with aggregate size. Binding inhibited both the aggregation reaction, specifically the elongation and reduced aggregate toxicity.

Lastly we have studied how nanobodies alter the aggregation of ɑS [41]. One widely explored therapeutic strategy for treatment of neurodegenerative disease is the use of antibodies, although a detailed molecular-level mechanism of the action of such species remains elusive. We characterized αS aggregation in the presence of two ɑS-specific single-domain antibodies (nanobodies), NbSyn2 and NbSyn87, which bind to the highly accessible C-terminal region of αS. We found that both nanobodies inhibit the formation of αS fibrils. Furthermore, we demonstrated that nanobody binding promotes a rapid conformational conversion from more stable oligomers to less stable oligomers of αS, leading to a dramatic reduction in oligomer-induced cellular toxicity. This is in contrast to our previous studies of Hsp70 or chaperones where binding did not alter the oligomer structure. This result suggests that the C-terminal region is more buried in the stable, beta sheet containing oligomers so that binding to this region by nanobodies causes conversion into less stable and less toxic oligomers. How much this effect is due to the smaller size of the nanobodies compared to conventional antibodies and how much to targeting the C-terminal region needs to be further investigated. However, it is an important finding since it provides a potential mechanism to rapidly convert toxic aggregates into less toxic and less stable aggregates, reducing the toxic load on neurons.

### 6.5 Thermodynamics of aggregate formation

There have been few studies of the thermodynamics of aggregate formation, in contrast to the large number of studies performed on the aggregation of the key proteins associated with neurodegenerative disease (Aβ, tau and αS). Even basic information about the critical aggregation concentration at which monomer is in equilibrium with fibrils has not been firmly established for these proteins. Furthermore, there is little literature on the cellular concentration of these proteins making it difficult to apply a quantitative approach to the problem of aggregate formation. There is also little information on the thermodynamics of oligomer formation, although this is more understandable given the technical issues in detecting small aggregates.

Multiple isoforms of aggregation-prone proteins are present under physiological conditions and have the propensity to assemble into co-oligomers with different properties from self-oligomers [42], but this process has not been quantitatively studied to date. We have investigated the Aβ peptide, associated with AD, and the aggregation of its two major isoforms, Aβ40 and Aβ42, using a statistical mechanical modelling approach in combination with test-tube single-molecule fluorescence measurements [21]. We found that at low concentrations of Aβ, corresponding to its physiological abundance, there is little free energy penalty in forming co-oligomers, suggesting that the formation of both self-oligomers and co-oligomers is possible under these conditions.

A model was used to predict the oligomer concentration and size at physiological concentrations of Aβ and suggests the mechanisms by which the ratio of Aβ42 to Aβ40 can affect cell toxicity [21]. An increased ratio of Aβ42 to Aβ40 raises the fraction of oligomers containing Aβ42, which can increase the hydrophobicity of the oligomers and thus promote deleterious binding to the cell membrane and increase neuronal damage. Our results suggest that co-oligomers are a common form of aggregate when Aβ isoforms are present in solution and may potentially play a significant role in AD.

The more complex in vivo environment is potentially affected by numerous extrinsic factors such as the presence of small molecules and proteins, lipid surfaces, altered pH or ionic strength. Therefore, the underlying assumption of thermodynamic equilibrium may not be correct. However, our model predicted the oligomer concentration observed in human CSF, of about 1 pM concentration at a total Aβ concentration of 1 nM. Even though the amount of Aβ42 in the CSF is generally observed to decrease in AD, our model predicts that this has little effect on the total oligomer concentration, because their population is largely dominated by Aβ40 oligomers. This may provide a simple explanation for why most diagnostic tests for AD to date based on detecting the Aβ oligomer concentration in CSF observe little significant difference between controls and AD patients.

### 6.6 Prion-like spreading

There is mounting evidence that suggests that the protein assemblies involved in various neurodegenerative diseases, including aggregated tau, are capable of self-sustaining amplification, allowing the propagation of pathological assemblies along connected brain cells, resulting in progression from restricted areas to large numbers of brain regions [[43], [44], [45]]. This process is commonly referred to as prion-like spreading, given its similarities to prion disease. The spreading requires that on entry into the cytoplasm of cells, tau assemblies can seed the aggregation of native monomers, thereby initiating the formation of additional aggregates, which can be released and spread to neighbouring cells. Both tau filaments from recombinant protein as well as filamentous material extracted from tau mouse models or AD brains have been shown to act as seeds in various model systems and initiate the spreading of tau pathology.

On a molecular level, to achieve propagation of protein aggregates through the brain, an amplification of seeds is required by fragmentation or surface templated aggregation. We characterised the aggregation kinetics of unlabelled full-length wild-type and mutant P301S tau using the luminescent conjugated oligothiophene (pFTAA) and TIRF imaging of the aggregates [46]. We found that the aggregate length increased rapidly over the first 24 h and then, once the monomer was largely used up, the length of the aggregates decreased over the next 4 weeks due to fragmentation.

This experiment provided direct evidence for fragmentation and hence amplification of tau aggregates *in vitro*. The elongation and fragmentation rate constants that were measured suggested that prion-like spreading could explain the rate of propagation of tau aggregates through the human brain in AD. According to this study, the time needed for one aggregate to double into two aggregates is of the order of several months to years in humans which means that aggregates must be present in neurons for this period of time. This provides a simple explanation of why propagation is via neurons, since they do not divide and cell division would dilute any aggregates formed.

This initial work has only been performed on 4 repeat tau. So it will be interesting to measure the elongation and fragmentation rate constants for 3 repeat tau to see how they compare with 4 repeat tau and also perform co-aggregation experiments with 3 and 4 - repeat tau. In AD, the aggregates formed contain both 3 and 4 repeat tau, while in tauopathies the aggregates can contain only 3 or 4 repeat tau. The structure of 3repeat tau fibrils from a patient with Pick's disease has recently been determined [47] and differs from the structure of the fibrils from an AD patient [48] supporting the concept that different prion strains have different fibrillar structure and hence would be expected to propagate at different rates.

### 6.7 In cells

Imaging the formation of aggregates in cells and which sub-cellular organelles they interact with is a significant advance. This should allow the molecular mechanism of aggregate induced toxicity to be probed and reveal what cellular mechanisms fail and lead to aggregate formation. We have combined DNA PAINT with an amyloid specific aptamer or aggregate specific antibody, and shown that this technique is able to detect and super-resolve a range of aggregated species, including those formed by αS and Aβ [13]. We used this method to image the endogenous protein aggregates formed in neurons with 40 nm spatial resolution. In our initial experiments we found that neuronal cells derived from a Parkinson's patient with 3 copies of the SNCA gene contain a larger number of protein aggregates than those from healthy control. We also used DNA PAINT to show that αS oligomers, added exogenously, bound specifically to ATP synthase, by performing simultaneous imaging using an antibody to ATP synthase [49]. This interaction was confirmed by the use of a proximity ligation assay. Similar experiments were performed with antibodies to an outer mitochondrial protein, TOMM20, and an inner mitochondrial membrane protein. These experiments show the potential of DNA PAINT imaging of aggregates in cells.

### 6.8 Characterising the real aggregates in humans

We have used the methods described here to perform an initial study of the soluble aggregates present in humanCSF from patients who are affected with AD. In these experiments, we measured the number of aggregates using Thioflavin T and their ability to permeabilise a lipid membrane [50]. We have also tested the effectiveness of a single-chain nanobody raised against the Aβ peptide, the extracellular chaperone Clusterin, and bapineuzumab (a humanized monoclonal antibody that was recently used in an unsuccessful AD drug trial) at reducing the Ca^2+^ influx induced by samples of CSF. We found that both control and AD CSF contained comparable numbers of soluble aggregates, unlike our study of Parkinson's disease CSF where there was a significant increase compared to controls [11]. In addition, both AD and control CSF caused detectable membrane permeabilisation and calcium ion entry. This could be blocked by Clusterin and a nanobody to Aβ, but the bapineuzumab was ineffective unless used at high concentrations. This study shows that toxic aggregates are present in CSF, providing a method to test potential therapies without costly clinical trials. The changes that take place during progression of AD are either more subtle in terms of aggregate composition or structure or that they are so diluted from the affected brain region that there are too small to be detectable when extracted using a lumbar puncture. If this is the case, it will be necessary to study samples derived from processed human brain tissue samples.

## 7 Future directions

Arguably one of the biggest problems in the field has been the difficulty in detecting and characterising the protein aggregates associated with neurodegenerative disease. This is due to their low concentration and high heterogeneity. The recent development of methods with single aggregate sensitivity solves this issue by opening up the possibility of characterising aggregates in cells, human brain tissue and biofluids such as CSF, and following the aggregation process in cells. More aggregate specific binding agents, such as conformational specific antibodies, will be needed to make it possible to better characterise the composition of the different aggregates to complement the measurement of their size, number and location.

Our lab is starting to use rationally designed antibodies for oligomers which allow the targeting of any region on the protein, without the need for it to be immunogenic [51]. An outstanding problem is to be able to identify which of the aggregates are toxic and by what mechanism. This can be done by measuring the toxicity of samples with a different combination of aggregates with different structures and correlating the different structures with the changes in toxicity. Alternatively, one could perform experiments on aggregates separated on the basis of a particular property such as size or mass. However, other sensitive toxicity assays need to be developed to complement the membrane permeabilisation assay, since aggregates can be toxic to cells by a number of different mechanisms. In principle, direct imaging of an aggregate binding to a labelled receptor would allow one to identify the both the toxic aggregate and the pathway, although this will require screening of all possible receptors. The ability to detect the aggregates in humans and measure their disease relevant biological activity should enable potential therapeutic strategies to be tested and optimised prior to costly clinical trials.

Overall one can hope, with some justifications, that the main tools are now in place to start to determine the key aggregate species responsible for the initiation and spreading of neurodegenerative disease and hence start to rationally design and test therapies that target these toxic species.
